# Supplementary material for: Genetic Mapping to Detect Stringent QTLs Using 1k-RiCA SNP Genotyping Platform from the New Landrace Associated with Salt Tolerance at the Seedling Stage in Rice
Source: Plants (Basel). 2022 May 26;11(11):1409. doi: 10.3390/plants11111409 (PMC9183132; doi:10.3390/plants11111409)
Supplement: Supplementary file 1 [file plants-11-01409-s001.zip › plants-1716434-supplementary.pdf]

Table S1: List of SNPs (**SNP ID**) using in the QTL analysis

**SNP Code**	**SNP ID**	**SNP Code**	**SNP ID**	**SNP Code**	**SNP ID**
C1	chr01_194844	C24	chr01_9164508	C47	chr01_16596849
C2	chr01_375814	C25	chr01_10100605	C48	chr01_17008280
C3	chr01_717702	C26	chr01_10202227	C49	chr01_17219764
C4	chr01_932866	C27	chr01_10645259	C50	chr01_17426109
C5	chr01_1044946	C28	chr01_10846090	C51	chr01_18102213
C6	chr01_1175585	C29	chr01_11250486	C52	chr01_18332445
C7	chr01_1532281	C30	SALTOL-AUS	C53	chr01_18501890
C8	chr01_2100471	C31	SALTOL-ARO	C54	chr01_19008526
C9	chr01_2277961	C32	chr01_12216652	C55	chr01_19308987
C10	chr01_2532252	C33	chr01_12335190	C56	chr01_20262103
C11	chr01_2726468	C34	chr01_12809255	C57	chr01_20418175
C12	chr01_2865232	C35	chr01_12941687	C58	chr01_20562598
C13	chr01_3236648	C36	chr01_13442034	C59	chr01_21011077
C14	chr01_3529691	C37	chr01_13777831	C60	chr01_22554065
C15	chr01_4167976	C38	chr01_13902629	C61	IRGSP1_C01_22891448
C16	chr01_4307004	C39	chr01_14375630	C62	chr01_23091103
C17	chr01_5064126	C40	chr01_14508449	C63	chr01_23240729
C18	chr01_5510350	C41	chr01_14937939	C64	chr01_23656773
C19	chr01_6130240	C42	chr01_15092392	C65	IRGSP1_C01_23735018
C20	chr01_6543217	C43	chr01_15393352	C66	chr01_24050118
C21	chr01_7068516	C44	chr01_15883941	C67	chr01_24722142
C22	chr01_7563923	C45	chr01_16113201	C68	chr01_26187514

C23	chr01_8760562	C46	chr01_16284866	C69	chr01_27046296
SNP Code	SNP Id.	SNP Code	SNP Id.	SNP Code	SNP Id.
C70	chr01_27330724	C93	chr01_37680628	C116	chr02_316859
C71	chr01_28142020	C94	chr01_37920337	C117	chr02_494373
C72	chr01_28478660	C95	DTY1-1_1	C118	chr02_629512
C73	chr01_28826738	C96	DTY1-1_2	C119	chr02_890901
C74	chr01_30569798	C97	chr01_38632196	C120	DTY2-2_1
C75	chr01_31396842	C98	DTY1-1_3	C121	MS-IR36
C76	chr01_32041358	C99	SCT1_1	C122	chr02_1082416
C77	chr01_32359998	C100	SCT1_2	C123	chr02_1380527
C78	chr01_32563460	C101	QSES1-2_1	C124	chr02_1487009
C79	chr01_32766139	C102	SCT1_3	C125	chr02_1660713
C80	chr01_33164035	C103	SCT1_4	C126	chr02_2008370
C81	chr01_33472174	C104	chr01_39548506	C127	chr02_2334960
C82	chr01_33695501	C105	QSES1-2_2	C128	DTY2-2_2
C83	chr01_33901317	C106	chr01_39955529	C129	chr02_2819095
C84	chr01_34201543	C107	QSES1-2_3	C130	chr02_2983451
C85	chr01_34363755	C108	QSES1-2_4	C131	chr02_3136771
C86	chr01_34508997	C109	chr01_40699488	C132	chr02_3477202
C87	chr01_35259107	C110	chr01_41020155	C133	chr02_3830712
C88	chr01_35589637	C111	chr01_41911439	C134	chr02_4047646
C89	chr01_35794891	C112	chr01_42425574	C135	chr02_4342883
C90	chr01_36150523	C113	chr01_42877440	C136	IRGSP1_C02_4549475
C91	chr01_36419842	C114	chr01_43074709	C137	chr02_4823175

C92	chr01_37274755	C115	chr02_103273	C138	DTY2-2_3
SNP Code	SNP Id.	SNP Code	SNP Id.	SNP Code	SNP Id.
C139	chr02_4930742	C162	chr02_19642635	C185	chr02_28778297
C140	chr02_5836334	C163	chr02_19981768	C186	chr02_29011747
C141	chr02_6373446	C164	chr02_20145859	C187	chr02_29415200
C142	chr02_6805305	C165	chr02_20824958	C188	chr02_29568762
C143	chr02_7238793	C166	chr02_21060669	C189	chr02_29718772
C144	chr02_7342223	C167	chr02_21832777	C190	chr02_30345735
C145	chr02_8019748	C168	chr02_22340836	C191	chr02_30530432
C146	chr02_8814718	C169	chr02_23073414	C192	chr02_31135814
C147	chr02_9570228	C170	IRGSP1_C02_23246863	C193	chr02_31403314
C148	chr02_9801099	C171	chr02_23396915	C194	chr02_31678155
C149	chr02_9975660	C172	chr02_23538743	C195	chr02_31914486
C150	HIS1	C173	chr02_23852677	C196	chr02_32101050
C151	chr02_10555263	C174	chr02_24168273	C197	chr02_32303539
C152	chr02_10874369	C175	chr02_24264653	C198	chr02_33035683
C153	chr02_11701123	C176	chr02_24772301	C199	chr02_34072964
C154	chr02_13962476	C177	chr02_24887320	C200	chr02_34425408
C155	chr02_16464872	C178	chr02_25193353	C201	chr02_34707946
C156	chr02_16919033	C179	chr02_25415397	C202	IRGSP1_C02_34852782
C157	chr02_17160895	C180	chr02_25949636	C203	chr02_34865754
C158	chr02_17497082	C181	chr02_26406867	C204	chr02_34868096
C159	chr02_17789129	C182	chr02_27548893	C205	chr02_35216781
C160	chr02_18370293	C183	chr02_28049479	C206	chr02_35401891

C161	chr02_18811812	C184	chr02_28211032	C207	chr02_35568365
SNP Code	SNP Id.	SNP Code	SNP Id.	SNP Code	SNP Id.
C208	chr02_35818319	C231	chr03_8532555	C254	chr03_17827706
C209	chr03_484812	C232	chr03_8711509	C255	chr03_18273682
C210	chr03_851785	C233	chr03_9026803	C256	chr03_18629240
C211	chr03_1270943	C234	chr03_9375374	C257	chr03_19667207
C212	DTY3-2-SWARNA_1	C235	chr03_9636224	C258	chr03_19929911
C213	DTY3-2-IR64_1	C236	chr03_9751213	C259	chr03_20664396
C214	DTY3-2-N22_1	C237	chr03_9878813	C260	chr03_21003404
C215	chr03_1481412	C238	chr03_10184356	C261	chr03_22050510
C216	chr03_1761412	C239	chr03_10304250	C262	chr03_22633713
C217	chr03_2151496	C240	chr03_10544320	C263	chr03_23037368
C218	chr03_2885423	C241	chr03_10798982	C264	chr03_23354868
C219	chr03_3382980	C242	chr03_11644944	C265	chr03_23851835
C220	chr03_3657643	C243	chr03_11851886	C266	chr03_24413888
C221	chr03_4047654	C244	chr03_12564300	C267	chr03_24682521
C222	chr03_4321231	C245	chr03_13224089	C268	chr03_25157655
C223	chr03_5109298	C246	chr03_13932387	C269	chr03_25259336
C224	chr03_5880211	C247	chr03_14169727	C270	chr03_25530777
C225	chr03_6283461	C248	chr03_14554651	C271	chr03_25833182
C226	chr03_6902921	C249	IRGSP1_C03_14933451	C272	chr03_26334305
C227	chr03_7200676	C250	chr03_15072791	C273	chr03_26456796
C228	chr03_7605347	C251	chr03_15365726	C274	chr03_26982172
C229	IRGSP1_C03_7752789	C252	GS3	C275	chr03_27275567

C230	chr03_7798537	C253	chr03_17307455	C276	chr03_27494934
SNP Code	SNP Id.	SNP Code	SNP Id.	SNP Code	SNP Id.
C277	chr03_27813704	C300	chr03_35824355	C323	chr04_7871436
C278	chr03_28851199	C301	chr03_36206765	C324	chr04_8217877
C279	chr03_29152266	C302	DTY4-1_1	C325	chr04_10077188
C280	chr03_29430694	C303	chr04_193676	C326	chr04_10468507
C281	chr03_30247826	C304	IRGSP1_C04_602842	C327	chr04_10991011
C282	chr03_30604896	C305	DTY4-1_2	C328	chr04_11645506
C283	chr03_31025197	C306	chr04_1022225	C329	chr04_11879947
C284	DTY3-1_2	C307	chr04_1136986	C330	chr04_12594753
C285	chr03_31190989	C308	chr04_1719865	C331	chr04_12665796
C286	DTY3-1_4	C309	DTY4-1_3	C332	chr04_12684621
C287	chr03_31427789	C310	chr04_3411505	C333	chr04_12711415
C288	chr03_31614921	C311	IRGSP1_C04_4319691	C334	chr04_12955347
C289	chr03_32063693	C312	chr04_4456304	C335	chr04_13551673
C290	chr03_32430702	C313	chr04_5111222	C336	chr04_13665093
C291	chr03_32577650	C314	chr04_5526487	C337	chr04_14229856
C292	chr03_32832500	C315	chr04_6027557	C338	chr04_14814591
C293	chr03_33080942	C316	chr04_6596219	C339	chr04_15389648
C294	chr03_33713486	C317	chr04_6913215	C340	chr04_15944977
C295	chr03_34275405	C318	BPH17_1	C341	chr04_16289829
C296	chr03_34476308	C319	BPH17_3	C342	chr04_16715200
C297	chr03_34678981	C320	BPH17_2	C343	chr04_17465113
C298	chr03_35093761	C321	chr04_7170237	C344	chr04_17666183

C299	chr03_35636092	C322	chr04_7304413	C345	chr04_18136052
SNP Code	SNP Id.	SNP Code	SNP Id.	SNP Code	SNP Id.
C346	chr04_18706317	C369	chr04_29533215	C392	chr05_1487229
C347	chr04_19123643	C370	chr04_29715617	C393	chr05_1832604
C348	chr04_19353709	C371	chr04_29881066	C394	chr05_2045112
C349	chr04_20507218	C372	chr04_30174083	C395	chr05_2291156
C350	chr04_21237749	C373	COLD1-JAP	C396	chr05_3092113
C351	chr04_21517348	C374	chr04_30438901	C397	chr05_3470087
C352	chr04_21864875	C375	chr04_30879875	C398	chr05_3817643
C353	chr04_22435296	C376	NAL1	C399	chr05_4205413
C354	chr04_22808095	C377	TBV1_2	C400	chr05_4589783
C355	chr04_23046635	C378	chr04_31509863	C401	chr05_4819475
C356	chr04_23248365	C379	chr04_32038496	C402	chr05_4953754
C357	chr04_23846299	C380	chr04_32189893	C403	GW5_1
C358	chr04_24061518	C381	chr04_33073892	C404	GW5_2
C359	RYMV1-5	C382	chr04_33448911	C405	chr05_6129755
C360	RYMV1-2	C383	chr04_34643455	C406	chr05_6544781
C361	chr04_25245272	C384	chr04_34925111	C407	chr05_6607675
C362	chr04_25437295	C385	chr04_35395197	C408	chr05_6833061
C363	chr04_26309089	C386	chr05_44806	C409	chr05_7195992
C364	chr04_26545849	C387	chr05_201193	C410	chr05_7328783
C365	chr04_26832471	C388	chr05_437499	C411	chr05_7706179
C366	chr04_27540988	C389	chr05_440644	C412	chr05_8080666
C367	chr04_28004788	C390	chr05_661832	C413	chr05_8384781

C368	chr04_28901886	C391	chr05_922530	C414	chr05_8585466
SNP Code	SNP Id.	SNP Code	SNP Id.	SNP Code	SNP Id.
C415	chr05_9712988	C438	chr05_20551103	C461	chr06_1521855
C416	chr05_10539124	C439	chr05_21494622	C462	WX-OP
C417	chr05_10886331	C440	chr05_22295097	C463	WX-INT
C418	chr05_11679660	C441	chr05_22711811	C464	chr06_1768006
C419	chr05_13325546	C442	chr05_23143060	C465	WX-A_GROUP
C420	chr05_13768744	C443	chr05_23661597	C466	WX-A-RC222
C421	IRGSP1_C05_13854594	C444	chr05_24090514	C467	chr06_1768998
C422	chr05_14412355	C445	chr05_25234430	C468	chr06_2430760
C423	chr05_14928105	C446	chr05_27086886	C469	chr06_2535102
C424	chr05_16262586	C447	chr05_27531788	C470	chr06_2783620
C425	chr05_16500527	C448	chr05_27627633	C471	chr06_2808467
C426	chr05_16752221	C449	chr05_27899049	C472	chr06_3140863
C427	chr05_16955234	C450	chr05_28101376	C473	chr06_3244503
C428	chr05_17012134	C451	chr05_28344771	C474	chr06_3431721
C429	chr05_17017106	C452	chr05_28772543	C475	chr06_3598843
C430	chr05_18405433	C453	chr05_29100308	C476	chr06_3632725
C431	chr05_18816269	C454	chr06_244274	C477	chr06_3721346
C432	chr05_19098650	C455	chr06_400753	C478	chr06_3951547
C433	chr05_19155333	C456	chr06_646915	C479	chr06_4076903
C434	chr05_19516545	C457	chr06_677288	C480	chr06_4641044
C435	chr05_19723342	C458	BPH32	C481	chr06_4757948
C436	chr05_19974373	C459	chr06_1272777	C482	chr06_5195334

C437	chr05_20331215	C460	chr06_1378891	C483	chr06_5707236
SNP Code	SNP Id.	SNP Code	SNP Id.	SNP Code	SNP Id.
C484	chr06_5894169	C507	chr06_16806254	C530	XA7_3
C485	chr06_6093491	C508	chr06_17076390	C531	chr06_28201282
C486	chr06_6508525	C509	chr06_17698448	C532	chr06_28723997
C487	SSIIA-3B	C510	chr06_19989362	C533	chr06_29056693
C488	chr06_7629011	C511	chr06_20462203	C534	chr06_29208264
C489	chr06_8153078	C512	chr06_21246829	C535	chr06_29416997
C490	chr06_8405025	C513	chr06_21491695	C536	chr06_30809492
C491	chr06_9222038	C514	chr06_21818860	C537	chr07_514293
C492	chr06_9836381	C515	chr06_22371041	C538	chr07_770090
C493	chr06_10006906	C516	chr06_24003856	C539	chr07_940832
C494	PI9_1	C517	TGW6	C540	chr07_1058718
C495	chr06_10418594	C518	chr06_25277863	C541	chr07_1195861
C496	chr06_10966744	C519	chr06_26139965	C542	chr07_1317761
C497	chr06_11131403	C520	chr06_26513610	C543	chr07_1432567
C498	chr06_11692013	C521	chr06_26938344	C544	chr07_1558687
C499	chr06_12989252	C522	chr06_27275515	C545	chr07_1865306
C500	chr06_13702743	C523	chr06_27364485	C546	chr07_2128881
C501	chr06_14006726	C524	chr06_27627615	C547	chr07_2331583
C502	chr06_14504992	C525	chr06_27761109	C548	chr07_4481788
C503	chr06_15358704	C526	XA7_1	C549	chr07_5442983
C504	chr06_15538142	C527	XA7_2	C550	chr07_5729056
C505	chr06_16304717	C528	chr06_28100960	C551	chr07_6029723

C506	chr06_16614854	C529	chr06_28107682	C552	chr07_7317122
SNP Code	SNP Id.	SNP Code	SNP Id.	SNP Code	SNP Id.
C553	chr07_8140854	C576	chr07_23087994	C599	chr08_2878924
C554	chr07_8320872	C577	AG3_1	C600	chr08_3212821
C555	chr07_8487862	C578	chr07_23535052	C601	chr08_3365127
C556	GHD7-4	C579	chr07_23785728	C602	chr08_3682006
C557	chr07_11290725	C580	AG3_2	C603	chr08_3866523
C558	chr07_13639513	C581	chr07_24194267	C604	chr08_4190544
C559	chr07_14729119	C582	chr07_24313817	C605	DTH8-IR24
C560	chr07_15340454	C583	chr07_24507157	C606	chr08_4511882
C561	IRGSP1_C07_16037152	C584	chr07_25339609	C607	chr08_4853081
C562	chr07_16313631	C585	chr07_25935801	C608	GM4_1
C563	chr07_17654305	C586	chr07_27021585	C609	chr08_5394572
C564	chr07_17877865	C587	chr07_27883987	C610	GM4_3
C565	chr07_18208798	C588	chr07_28187994	C611	GM4_4
C566	chr07_18503646	C589	chr07_28409912	C612	chr08_5634800
C567	chr07_18689329	C590	chr07_29062014	C613	PI33_1
C568	chr07_18895399	C591	chr08_213580	C614	chr08_6269190
C569	IRGSP1_C07_19408599	C592	chr08_658032	C615	PI33_2
C570	chr07_20514892	C593	chr08_811201	C616	PI33_3
C571	chr07_21135004	C594	chr08_1088007	C617	chr08_7854002
C572	chr07_21539524	C595	chr08_1198055	C618	chr08_7950638
C573	TSV1_UM	C596	chr08_1836529	C619	chr08_8386031
C574	chr07_22277144	C597	chr08_2208785	C620	chr08_8580913

C575	chr07_22593213	C598	chr08_2673447	C621	chr08_8703041
SNP Code	SNP Id.	SNP Code	SNP Id.	SNP Code	SNP Id.
C622	chr08_8897653	C645	chr08_15439243	C668	chr08_25467581
C623	chr08_9289473	C646	chr08_16618829	C669	chr08_25757847
C624	chr08_9769758	C647	chr08_16799413	C670	PGWC8-2
C625	chr08_10105233	C648	chr08_17319167	C671	chr08_26448560
C626	chr08_10188041	C649	chr08_17618902	C672	XA13_2
C627	chr08_10358484	C650	chr08_18108496	C673	XA13_1
C628	IRGSP1_C08_10441781	C651	chr08_18221884	C674	chr08_26709228
C629	chr08_11248949	C652	chr08_18552226	C675	chr08_26755059
C630	chr08_11370680	C653	chr08_18719247	C676	chr08_26898822
C631	chr08_11565636	C654	chr08_19351882	C677	chr08_27039158
C632	chr08_11727432	C655	chr08_19833397	C678	IRGSP1_C08_27133000
C633	chr08_11888511	C656	chr08_20053642	C679	chr08_27282645
C634	chr08_12145970	C657	chr08_21011651	C680	chr08_27563586
C635	chr08_12421145	C658	chr08_21253397	C681	chr08_28125942
C636	chr08_12723619	C659	chr08_21667240	C682	chr09_148594
C637	chr08_13017690	C660	chr08_22252502	C683	chr09_496013
C638	chr08_13354771	C661	chr08_22776414	C684	chr09_591085
C639	chr08_13741571	C662	chr08_23713744	C685	chr09_1098249
C640	chr08_13950899	C663	chr08_24125399	C686	chr09_1467951
C641	chr08_14184612	C664	chr08_24205833	C687	chr09_2096551
C642	chr08_14676962	C665	chr08_24753844	C688	chr09_2298381
C643	chr08_14799421	C666	IRGSP1_C08_24853648	C689	chr09_2380203

C644	chr08_14986111	C667	WFP1	C690	chr09_2930321
SNP Code	SNP Id.	SNP Code	SNP Id.	SNP Code	SNP Id.
C691	chr09_3357568	C714	chr09_10757351	C737	chr09_21667022
C692	chr09_3603443	C715	chr09_11237877	C738	chr09_21885499
C693	chr09_3934768	C716	chr09_11746684	C739	chr09_21896910
C694	chr09_4136552	C717	chr09_11848827	C740	chr09_22076185
C695	chr09_4347203	C718	AG1_1	C741	chr10_146531
C696	chr09_5008706	C719	chr09_12029697	C742	chr10_790044
C697	chr09_5305969	C720	chr09_12326525	C743	chr10_1562931
C698	chr09_5812738	C721	chr09_14920092	C744	chr10_2129424
C699	chr09_5922125	C722	chr09_15322775	C745	chr10_2258418
C700	chr09_6093984	C723	chr09_15429734	C746	chr10_3358419
C701	IRGSP1_C09_6205891	C724	DEP1-1	C747	chr10_3400212
C702	chr09_6252407	C725	DEP1-2	C748	chr10_4019997
C703	chr09_6796269	C726	chr09_16616509	C749	chr10_4281052
C704	chr09_6913547	C727	chr09_17213519	C750	chr10_5257224
C705	chr09_7116276	C728	chr09_17447522	C751	chr10_5488256
C706	chr09_7280339	C729	chr09_17707080	C752	chr10_5826161
C707	chr09_8511436	C730	chr09_19322095	C753	chr10_6648158
C708	chr09_8851945	C731	chr09_19683788	C754	chr10_6798684
C709	chr09_9252104	C732	chr09_20138174	C755	chr10_7120214
C710	chr09_9626056	C733	chr09_20682114	C756	chr10_7397104
C711	chr09_9945591	C734	chr09_21148430	C757	chr10_7530092
C712	chr09_10119114	C735	chr09_21348882	C758	chr10_8404652

C713	chr09_10324014	C736	chr09_21612758	C759	chr10_8795028
SNP Code	SNP Id.	SNP Code	SNP Id.	SNP Code	SNP Id.
C760	chr10_9095431	C783	chr10_18852690	C806	chr11_8601502
C761	chr10_10476379	C784	chr10_19025553	C807	chr11_8786491
C762	chr10_10755400	C785	chr10_19217152	C808	chr11_9183139
C763	chr10_11195773	C786	chr10_19378143	C809	chr11_9413614
C764	chr10_11664750	C787	chr10_19421322	C810	chr11_9561302
C765	chr10_11918679	C788	chr10_19678268	C811	chr11_9933184
C766	IRGSP1_C10_12035291	C789	chr10_19899160	C812	chr11_10741559
C767	chr10_12145295	C790	chr10_21338558	C813	chr11_11715177
C768	chr10_12658334	C791	chr10_21596449	C814	chr11_12142966
C769	chr10_12780901	C792	chr11_256688	C815	chr11_12583306
C770	chr10_13551338	C793	chr11_650148	C816	chr11_13020856
C771	chr10_13778768	C794	chr11_2393690	C817	chr11_13926471
C772	chr10_14086902	C795	chr11_2875870	C818	chr11_14737362
C773	chr10_14640458	C796	chr11_3516253	C819	chr11_15447862
C774	chr10_14777004	C797	chr11_3802930	C820	chr11_15638505
C775	chr10_15824876	C798	chr11_4783262	C821	chr11_15850522
C776	chr10_16229530	C799	chr11_5615885	C822	chr11_16284824
C777	chr10_16742656	C800	chr11_5852961	C823	chr11_17047762
C778	chr10_17397576	C801	chr11_6088667	C824	chr11_17292835
C779	chr10_18098744	C802	chr11_6472086	C825	chr11_17489208
C780	chr10_18302926	C803	chr11_7542820	C826	chr11_17697337
C781	chr10_18583084	C804	chr11_8083891	C827	chr11_17908099

C782	RF4	C805	chr11_8247118	C828	STV11
SNP Code	SNP Id.	SNP Code	SNP Id.	SNP Code	SNP Id.
C829	SWEET14_PRO1	C852	chr11_23023708	C875	XA26_1
C830	SWEET14_2	C853	chr11_23080452	C876	XA4_2
C831	chr11_18290104	C854	chr11_23083051	C877	chr11_28625316
C832	chr11_18936070	C855	chr11_23231455	C878	chr11_28966693
C833	chr11_18993024	C856	IRGSP1_C11_23731426	C879	chr12_350921
C834	chr11_19648542	C857	chr11_24086107	C880	chr12_2324105
C835	chr11_19898835	C858	chr11_24100905	C881	chr12_2651274
C836	chr11_20074439	C859	chr11_24585862	C882	chr12_3086293
C837	chr11_20239426	C860	chr11_25135188	C883	chr12_3217524
C838	chr11_20647651	C861	PI54_5	C884	chr12_3285571
C839	chr11_20837771	C862	chr11_25279481	C885	chr12_3544726
C840	chr11_21190115	C863	chr11_25432067	C886	chr12_4141420
C841	chr11_21446099	C864	chr11_25811598	C887	chr12_4433511
C842	chr11_21689303	C865	chr11_26300403	C888	chr12_4554103
C843	chr11_21691103	C866	IRGSP1_C11_27391141	C889	chr12_5041059
C844	chr11_22044151	C867	IRGSP1_C11_27571141	C890	chr12_7114076
C845	XA23_2	C868	chr11_27603799	C891	chr12_7445812
C846	IRGSP1_C11_22303236	C869	chr11_27817978	C892	chr12_7917509
C847	chr11_22440795	C870	PIK_2	C893	chr12_8070782
C848	chr11_22453819	C871	PIK_1	C894	chr12_8240608
C849	chr11_22562351	C872	PIKH_2	C895	chr12_8794364
C850	PB1_1	C873	PIKH_1	C896	chr12_8913217

C851	chr11_22912310	C874	chr11_27994133	C897	chr12_9120135
SNP Code	SNP Id.	SNP Code	SNP Id.	SNP Code	SNP Id.
C898	chr12_9540170	C916	chr12_15960393	C934	chr12_23108164
C899	chr12_10051752	C917	IRGSP1_C12_15966147	C935	chr12_23190179
C900	chr12_10469146	C918	chr12_16287347	C936	chr12_23387696
C901	chr12_10509878	C919	chr12_16591134	C937	chr12_23828922
C902	chr12_11257069	C920	chr12_16990670	C938	chr12_24088209
C903	chr12_11720114	C921	chr12_17443323	C939	chr12_24145303
C904	chr12_12075622	C922	DTY12-1_2	C940	chr12_24317071
C905	chr12_12509917	C923	chr12_17571574	C941	chr12_24417433
C906	chr12_12904225	C924	chr12_18003414	C942	chr12_24705450
C907	chr12_13248798	C925	chr12_18881059	C943	chr12_24913573
C908	chr12_13764151	C926	chr12_19161731	C944	chr12_25490919
C909	chr12_14060565	C927	chr12_19522102	C945	chr12_25756224
C910	chr12_14489210	C928	chr12_19578931	C946	chr12_26259494
C911	chr12_14599449	C929	chr12_19829588	C947	chr12_26463351
C912	chr12_14835375	C930	chr12_21446549	C948	chr12_26665728
C913	chr12_14936674	C931	chr12_21731719	C949	chr12_27023424
C914	chr12_15140411	C932	chr12_22600624	C950	chr12_27304351
C915	chr12_15342492	C933	chr12_23066809		
